# Editorial: New Insights Into and Interventions of the Persistent Immune Responses in Chronic Graft Rejection

**DOI:** 10.3389/fimmu.2021.759189

**Published:** 2021-09-03

**Authors:** Dejun Kong, Caigan Du, Hao Wang

**Affiliations:** ^1^Department of General Surgery, Tianjin Medical University General Hospital, Tianjin, China; ^2^Tianjin General Surgery Institute, Tianjin, China; ^3^Organ Transplantation Center, Tianjin First Central Hospital, Tianjin, China; ^4^School of Medicine, Nankai University, Tianjin, China; ^5^Department of Urologic Sciences, the University of British Columbia, Vancouver, BC, Canada; ^6^Immunity and Infection Research Centre, Vancouver Coastal Health Research Institute, Vancouver, BC, Canada

**Keywords:** transplantation, chronic graft rejection, innate immunity, organ preservation, treatment

## Introduction

Solid organ transplantation is the best therapeutic option for the patients who are suffered from end-stage organ failure. In practice, the organ transplantation is mainly used as either a form of life-saving or life-enhancing. However, due to the donor organ shortage crisis, not every patient on the waiting list can receive a donor organ. During the past few decades, with the great development of surgical skills, post-operative monitoring and care, and immunosuppressive agents, the short-term graft survival has been significantly improved after organ transplantation, indicated by as high as 95% of survival rate (1). Nevertheless, the incidence of long-term graft insufficiency or dysfunction remains very high (49.7% from the marginal donors and 34.1% from living donors) (2), which becomes one of the biggest obstacles to achieving the long-term function of a transplanted organ. There are several immune and non-immune factors involving in the pathogenesis of chronic allograft rejection (CAR), including donor’s individual circumstances, ischemia reperfusion injury (IRI), innate and adaptive immunity and so on. This Research Topic gathers different contributions highlighting the recent pre-clinical and clinical researches regarding the new insights into the CAR.

## Innate Immunity, Macrophage and CAR

A diversity of innate immune cells has received much attention towards the understanding of CAR. Increasing evidence focuses on the profound roles of innate immune cells, such as NK cells, macrophages, and neutrophils, in the late phase change of the allografts. Emerging attention has been paid to the intragraft macrophage infiltration in the immunopathological characteristics of CAR. It has been well established that M1-like macrophages are inclined to produce pro-inflammatory cytokines TNF-α, IL-6, and IL-1β, mediating IRI in the early stage after transplantation. As pro-/anti- inflammatory activation and tissue repair co-exist, grafts may undergo dynamic change. Failing to control or prevent acute inflammation may result in chronic inflammation, attributing to establishment of allograft inflammatory fibrosis and gradual deterioration in functions.

Zhang et al. have reviewed the roles of macrophages as a pivotal part of innate immunity in CAR. In their review, the diverse functions of M1 and/or M2 macrophages that drive different pathophysiological mechanisms in the acute or CAR are elaborated. Importantly, despite the fact that the predominated effects of adaptive antibody-mediated rejection on the loss of the grafts, the innate-immune-mediated fibrotic rejection triggered by the macrophages should not be neglected. The pathogenesis of the tissue fibrosis is a shared and central part of numerous pathologies, including autoimmunity, metabolic disorders, and graft rejection. The M2 macrophages not only contribute to anti-inflammation in the very early stage of graft survival, but also promote fibrotic processes *via* producing TGF-β1 and VEGF. This review provides us with better insights into the crosstalk between the macrophages and CAR.

## Biomarkers for CAR

As described above, there is an emerging pivotal role of the macrophages in the pathogenesis of CAR (3). Macrophage infiltration after one year post-transplantation has been demonstrated to be significantly associated with graft dysfunction and fibrosis. Furthermore, CD68^+^CD163^+^ macrophages (M2 macrophage) tend to increase in grafts with chronic antibody-mediated rejection as compared those with acute antibody-mediated rejection and T cell-mediated rejection, suggesting that these macrophages could promote the chronic progressive injury (4).

Zhan et al. have reported that PC3-secreted microprotein (PSMP) functions as a newly identified chemokine that could interact with C-C motif chemokine receptor 2 so as to mediate macrophage infiltration in local tissue, which may recruit the macrophages and promote the intimal arteritis, resulting in allograft lost in cABMR. This study may indicate PSMP may be a potential histopathological diagnostic biomarker and therapeutic target for kidney transplant rejection. However, whether PSMP plays the same role as in other organ transplants is not clear.

## Improper Organ Preservation Leads to Allograft Rejection

At present, the organ donors for organ transplantation around the world are mainly from Donation after Cardiac Death (DCD) donors (5). However, the DCD marginal organs are at a high risk of short-term or long-term graft loss, depending on the donor’s individual circumstances and organ preservation post-harvest. It has been known that in both humans and animal models, transplants from DCD with a longer ischemia time are less likely to survive long-term and undergo chronic rejection as compared with those from living donors, which remains an obstacle to using organ transplantation as a common therapy around the world. Nishi et al. for the first time have reported that hydrogen benefits long-term prognosis of kidney transplants in a minipig model of the ischemic kidney transplantation and can inhibit chronic rejection response under a strict multi-immunosuppressant protocol.

## Novel Strategies to Prevent CAR

### Potential Therapeutic Targets Against CAR

Autophagy is a cellular physiological process responsible for protein and organelle degradation as a mechanism of cytoprotection against stress (6). Endothelial‐to‐mesenchymal transition (EndMT) plays an important role in the development of the fibrosis following kidney transplantation. Gui et al. have shown that the autophagic activity temporarily increases at the early stages of allograft transplantation, and it gradually decreases as ATG16L declines. ATG16L-dependent autophagy, as a cytoprotective process, could attenuate the transplanted kidney interstitial fibrosis *via* the regulation of the EndMT induced by IL-1β, IL-6 and TNF-α.

Zhang et al. have reported an interesting phenomenon indicating that ST2 deficiency in either allografts or recipients oppositely affects the cardiac allograft vasculopathy/fibrosis *via* differentially altering immune cells infiltration, suggesting that interrupting IL-33/ST2 signaling locally or systematically in heart transplantation may benefit the prevention of cardiac allograft vasculopathy. Nian et al. have also demonstrated that treatment with αIL-21R shifts the Tfh/Tfr balance toward DSA inhibition, which means that αIL-21R may be a potential therapeutic agent to prevent the chronic antibody-mediated rejection in organ transplantation. Finally, Li et al. have reported that mTOR deficiency enhances the immunosuppressive function of M-MDSCs and prolongs mouse cardiac allograft survival.

### Traditional Chinese Medicine and CAR

To suppress the host immune response to the graft and to improve the recipient’s tolerance to the transplant remain a primary focus in organ transplantation. Traditional Chinese medicine is an indispensable part in the health care in China. During the past decades, the extracts of Chinese medical herbs and their derivatives have been used in the treatment of immune- and non-immune-mediated diseases. Yang et al. have reported that artemisinin, a well-known anti-malaria agent, can effectively inhibit multiple lymphocytes, resulting in prolongation of cardiac allograft survival in a rat model of cardiac allotransplantation, and Zhou et al. have showed that curcumin, a natural polyphenol compound extracted from turmeric alleviates IL-6–dependent EndMT by promoting autophagy *in vitro* and *in vivo* in a rat model of kidney transplantation.

### Mesenchymal Stromal Cells and CAR

Mesenchymal stromal cell (MSC)-based therapy emerges as an effective method for immunomodulation after transplantation (7). MSCs are adult multipotent stem cells with potent immune regulation and tissue regeneration potential. Numerous studies have demonstrated that MSCs could induce long-term allograft tolerance *via* releasing cytokines, chemokines, and extracellular vesicles and so on (8).

Qin et al. have demonstrated that melatonin can synergize with MSCs in the inhibition of chronic allograft vasculopathy in a mouse model of heterotopic aortic transplantation *via* strengthening immunomodulatory effects of MSCs. Zheng et al. review the recent development of MSCs-derived exosomes in preventing the organ transplant rejection, which may provide new strategies for improving the long-term prognosis of organ transplantation patients. Surprisingly, Wei et al. have conducted a clinical trial to confirm the clinical efficacy of MSCs. In their clinical trial, kidney recipients with biopsy-proven chronic active antibody-mediated rejection (cABMR) are divided into Regimen 1 (n = 8, a dose of 1.0 × 10^6^ cells/kg monthly for four consecutive months) and Regimen 2 (n = 15, a dose of 1.0 × 10^6^ cells/kg weekly during four consecutive weeks). Contemporaneous cABMR patients who did not receive Bone marrow-derived-MSCs are retrospectively used as a control group (n = 30). Kidney allograft recipients with cABMR are tolerable to BM-MSCs, and the immunosuppressive drugs combined with intravenous BM-MSCs can delay the deterioration of allograft function, probably by decreasing DSA level and reducing DSA-induced injury.

## Conclusion

We hope that this Research Topic could provide a different perspective towards a further understanding of CAR and novel therapeutic strategies for the prevention of the CAR in the post-transplant care in the near future.

## Author Contributions

DK: first draft of manuscript writing. CD: manuscript editing. HW: financial support, administrative support, manuscript writing, final revision and approval of manuscript. All authors contributed to the article and approved the submitted version.

## Funding

This work was supported by grants to HW from National Natural Science Foundation of China (No. 82071802), Li Jieshou Intestinal Barrier Research Special Fund (No. LJS_201412), and Natural Science Foundation of Tianjin (No. 18JCZDJC35800)

## Conflict of Interest

The authors declare that the research was conducted in the absence of any commercial or financial relationships that could be construed as a potential conflict of interest.

## Publisher’s Note

All claims expressed in this article are solely those of the authors and do not necessarily represent those of their affiliated organizations, or those of the publisher, the editors and the reviewers. Any product that may be evaluated in this article, or claim that may be made by its manufacturer, is not guaranteed or endorsed by the publisher.
